# Environmental factors associated with freshwater recreational water quality in Niagara Region, Ontario, Canada: A path analysis

**DOI:** 10.1017/S0950268821002120

**Published:** 2021-09-22

**Authors:** J. Johanna Sanchez, Ian Young, Cole Heasley, Jeremy Kelly, Anthony Habjan, Ryan Waterhouse, Jordan Tustin

**Affiliations:** 1 School of Occupational and Public Health, Ryerson University, Toronto, Ontario, Canada; 2 Niagara Region Public Health, Thorold, Ontario, Canada

**Keywords:** Epidemiology, food safety, food-borne zoonoses, meta-analysis, systematic review

## Abstract

*Escherichia coli* concentration levels in recreational water are used by beach managers to evaluate the risk of gastrointestinal illness among beachgoers. We examined the relationship between specific environmental factors and *E. coli* concentration in recreational beaches in the Niagara Region. We analysed *E. coli* geometric means collected from eight beaches from two of the Great Lakes in the Niagara Region in Ontario, between 2011 and 2019. We applied path analysis to evaluate the relationship between the environmental factors and *E. coli* concentrations, including whether effects were direct or indirect via a mediator. Turbidity was found to be an important mediator for the indirect effect of environmental variables overall and in beach-specific models. Rainfall and streamflow had a positive indirect effect on *E. coli* via turbidity and a direct effect in five out of seven beach models. Streamflow was also a mediator for the indirect effect of previous day air temperature in five out of seven models. In three subset models, outfall *E. coli* concentration was a mediator for the effect of the environmental factors. Using a novel methodological approach, this study identifies important relationships and pathways that predict beach *E. coli* concentration in freshwater beaches located on two of the Great Lakes.

## Introduction

Poor recreational water quality, as indicated by a high concentration of pathogens, represents a risk of gastrointestinal illness for those engaging in water activities such as swimming [1, 2]. In Canada, beach water quality in freshwater bodies is regularly monitored by measuring faecal indicator bacteria (FIB) levels, most commonly *Escherichia coli (E. coli)*, as a surrogate for the presence of enteric pathogens and risk of gastrointestinal illness [3]. The Guidelines for Canadian Recreational Water Quality recommend a geometric mean concentration of less that 200 colony-forming units (CFU)/100 ml of *E. coli* as a threshold level suggesting an acceptable, low risk of gastrointestinal illness [3]. In the Niagara region, beaches along Lake Ontario and Lake Erie are monitored regularly for *E. coli* levels; however, the culture-based laboratory methods used in Ontario can require 18–24 h for results to be available [4]. As such, decisions about beach posting status (i.e. if the beach water is unsafe for swimming) are based on data from the previous day. This delay could result in a risk of illness to the swimmers as changing environmental conditions could result in a difference in water quality within hours [5].

Freshwater beach water *E. coli* concentrations are influenced by multiple environmental factors [6–8]. Rainfall is regularly reported as one of the most important predictors of freshwater lake quality, in predictive models, with increased rainfall resulting in higher microbial concentrations [6, 9]. Conversely, increased solar radiation has been associated with decreased *E. coli* counts [7]. This relationship is also affected by lake level, wave height and turbidity [7]. Turbidity, while not regularly collected as part of environmental monitoring at many public health units in general, has been increasingly studied as an important predictor for FIB, including *E. coli* [8].

The factors affecting *E. coli* concentrations in beach water can directly impact public health management strategies and policies. Research on freshwater beaches considers the unique characteristics that may influence the factors associated with the presence of FIB in freshwater. For example, *E. coli* is known to survive longer in freshwater and fresh waters lack the salinity and tidal cycles present in marine water [7]. Inland freshwater beaches require focused research and analysis in order to appropriately inform beach water monitoring programmes in making more timely decisions. Currently, there is a limited body of such research in the Ontario and Canadian context.

The environmental drivers of *E. coli* concentration may not act directly or independently of each other. Understanding the complex pathways of the relationships between the factors and *E. coli* could help direct water quality improvement efforts. Path analysis, is a powerful method that allows for gaining insight of the cause−effect relationships between variables, allowing for further understanding of complex relationships within interactions webs. Using this method, we are able to estimate the direct, indirect and total effects of factors on outcome variables [10].

The unique geography of Niagara Region, Ontario allows for the opportunity to examine freshwater beaches located along two of the Great Lakes. In addition, the management of beach water quality is of significant public health importance, with an extensive beach monitoring programme in place for the many popular beaches. Using daily water quality sampling data collected by the local health unit linked to publicly available federal and provincial environmental data, this study applies path analysis methodology to examine the key environmental factors of *E. coli* concentration at eight popular beaches.

## Methods

### Study area

**Fig. 1.**
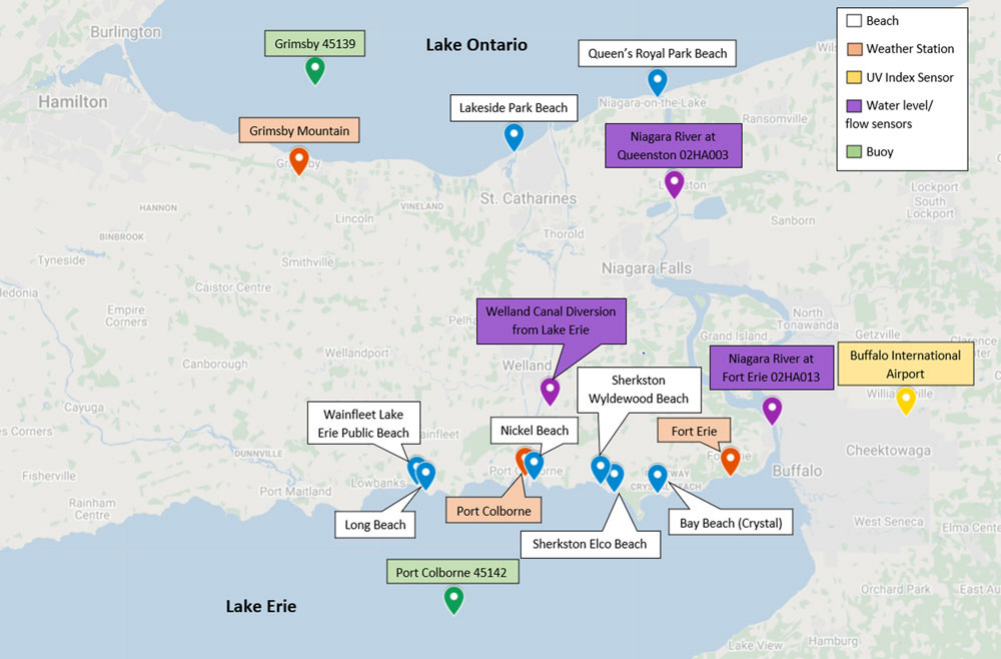
Selected beaches and climate stations in Niagara Region, 2011–2019.

Eight beaches monitored by Niagara Region Public Health were selected for the study based on the availability of regular water sampling data (Fig. 1). Two beaches are located along Lake Ontario and six along Lake Erie. The region has a total area of 1852 km^2^ and a population of 427 421 [11]. Two major water systems traverse the region in a northward direction, connecting Lake Erie to Lake Ontario: the Niagara River, which contains Niagara Falls and the Welland Canal.

### Water quality data

Samples for *E. coli* collected during the recreational season (May to September) from 2011 to 2019 were obtained by Niagara Region Public Health. Beach water samples were collected six times a week at seven of the participating beach study sites and four times a week at the remaining beach (Queen's Royal). Sample collection took place between 7 and 10 AM each day at knee to waist depth, 15–30 cm below the surface of the water, from five pre-specified sampling locations at each beach, following recommended provincial guidelines [12]. Water samples were centrally processed at a Public Health Ontario laboratory within one calendar day of collection using an accredited modified membrane filtration method [13]. The daily *E. coli* geometric mean for each beach was calculated by the public health unit based on the laboratory results of the five samples collected. In addition, water outfall samples were collected from stormwater runoff at four participating sites on a weekly basis and processed in the same manner. To address the skew of both the *E. coli* geometric mean and outfall *E. coli* values, log transformations were used to satisfy linear assumptions, prior to data analysis.

### Environmental data

Total daily precipitation (mm) and maximum, minimum and mean air temperature (°C) data were obtained from the Canadian Government's Environment and Natural Resources weather station historical data repository [14]. Three weather stations in the Niagara Region were selected based on completeness of data during the study period 2011–2019: Grimsby Mountain, Port Colborne and Fort Erie. Beaches were linked to a weather station based on lake location and proximity to the station (Supplementary Table S1). Wave height (m) and wind speed (knots) were collected from Environment Canada buoy station historical data [14]. Lake Ontario sites were linked to buoy 45 159, while sites located on Lake Erie were linked with buoy 45 142 (Supplementary Table S1, Fig. 1). Stream discharge data were collected from sensors located mid-way through the Niagara River and Welland Canal and publicly available on Environment Canada's streamflow historical data repository [14]. Sites were linked to sensor data based on proximity to the river or canal. Ultraviolet (UV) radiation data were collected from the closest station collecting these data, the U.S National Oceanic and Atmospheric Administration Weather Service station located in Buffalo, New York, United States [15]. Turbidity, measured in nephelometric turbidity units (NTU), was collected by Niagara Region Public Health as part of their water sampling collection process, at the third (middle-most) sampling site at each beach. Testing of the water samples for turbidity took place on-site using a Hach 2100P Turbimeter. To adjust the scale of the variables and to address skew of the data, turbidity and stream discharge were log transformed prior to analysis.

### Statistical analysis

We applied path analysis methodology, a powerful statistical method for examining causal patterns among variables, which is useful in understanding the influence of variables on one another [16]. A hypothesised conceptual path model (Supplementary Fig. S1) was developed based on available literature addressing the environmental factors influencing water quality, as measured by *E. coli*. This model featured the potential pathways between the environmental factors and the outcome variable, *E. coli* concentration, as well the intervariable relationships. Multicollinearity diagnostics ensured variables met the assumption of independence [17]. Variables were not included in the model if the variance inflation factor (VIF) exceeded 0.80. A multilevel approach was not required for this analysis as exploratory linear mixed effects modelling, including beach as a random effect, did not identify significant clustering of the *E. coli* values at the beach level.

To examine the temporal relationship between environmental conditions and the *E. coli* concentration, we examined values of the previous day for mean stream discharge, mean wave height and mean UV index. Air temperature was also captured as a previous day mean instead of a max and min, as it was expected that these values would be reflected in changing daily mean. We included same-day values of turbidity and streamflow. As we expect *E. coli* concentration on the previous day to be associated with the current day concentration, this previous day value was also included in the model. A cumulative rainfall variable was generated as the 2-day sum of precipitation (mm), preceding the day of collection of the water sample. All variables in the model were continuous. The pathways from these variables to our outcome variable, log_10_ *E. coli*, were hypothesised to operate via a direct relationship or indirectly via a mediator, such as turbidity, stream discharge and outfall (at applicable beaches). Specific indirect effects for each antecedent-mediator-outcome path were calculated.

Goodness of fit for each model was assessed by chi-square statistics, comparative fit index (CFI), Tucker–Lewis index (TLI) and root mean square error approximation (RMSEA). The models were considered to have a very good fit if the CFI and TLI were above 0.95 and RMSEA was below 0.05 [18]. Akaike information criterion (AIC) and Bayesian information criterion (BIC) were used to compare and select between models in the model-building phase. Analyses were carried out using Stata version 14.0 using the sem command.

## Results

### Descriptive data

**Fig. 2.**
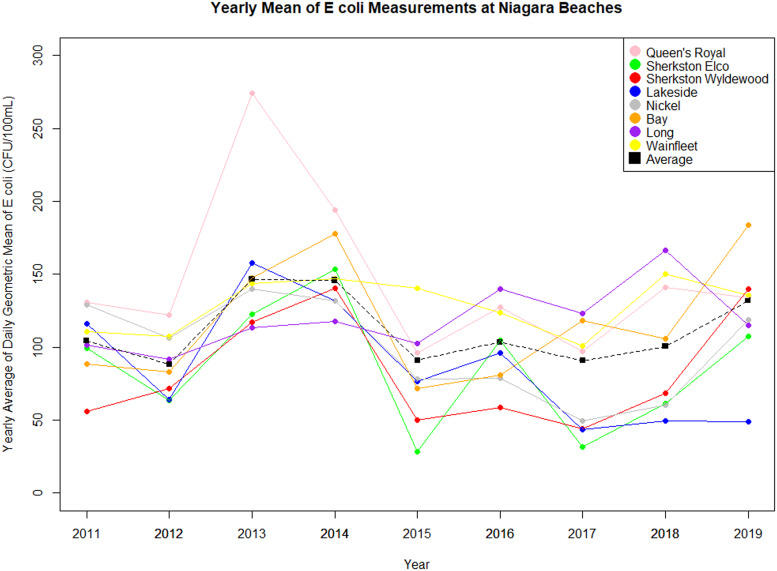
Mean annual *E. coli* geometric mean at Niagara Region Beaches, 2011–2019.

A total of 5149 observations from eight beach sites in the Niagara Region were included in the analyses and linked with daily environmental factor values (Supplementary Table S2). Overall geometric *mean E.* coli levels by beach are presented in Figure 2 and Supplementary Table S3. The highest annual geometric mean values during the study period were in 2013 and 2014. Queen's Royal beach had the highest overall, with the highest annual *E. coli* geometric mean of 274 CFU/100 ml in 2013. A subset of 1157 outfall observations were collected from Bay Beach, Long Beach, Queen's Royal beach and for some years at Nickel Beach (Supplementary Table S2). The highest outfall *E. coli* values were seen at Long Beach.

Beach postings are presented in Supplementary Table S4, overall and by beach site. Queen's Royal Beach had the highest proportion of beach days posted as unsafe for swimming overall and the highest annual proportion of days posted in 2013 (54%), which was also the year Queen's Royal Beach had the highest geometric mean. Lakeside Beach was posted as unsafe for swimming for most of 2019 due to high water levels in Lake Ontario that resulted in submersion of most of the beach in the lake. Unsurprisingly, most other beach postings throughout the study period were associated with high *E. coli* levels, with few resulting from algae, rainfall and other reasons (Supplementary Table S5). Other reasons were due mainly to visible debris.

Annual environmental factor values are summarised in Supplementary Table S6. Rainfall was presented as an annual cumulative value, while all other variables were presented as a mean or median of daily values. The highest rainfall for the study period was reported in 2018 with 513 mm of rain, followed by 2013 with 471 mm of rain. The highest mean air temperature was in 2018 (24.3 °C), followed by 2013 (22.8 °C). The highest seasonal turbidity was in 2019 at 6.5 Nt\TU (Supplementary Table S6). Of note is also the high stream discharge value in the Niagara River, with the highest daily mean reported in 2019 with a value of 7850 m^3^/s.

### Path analysis

**Fig. 3.**
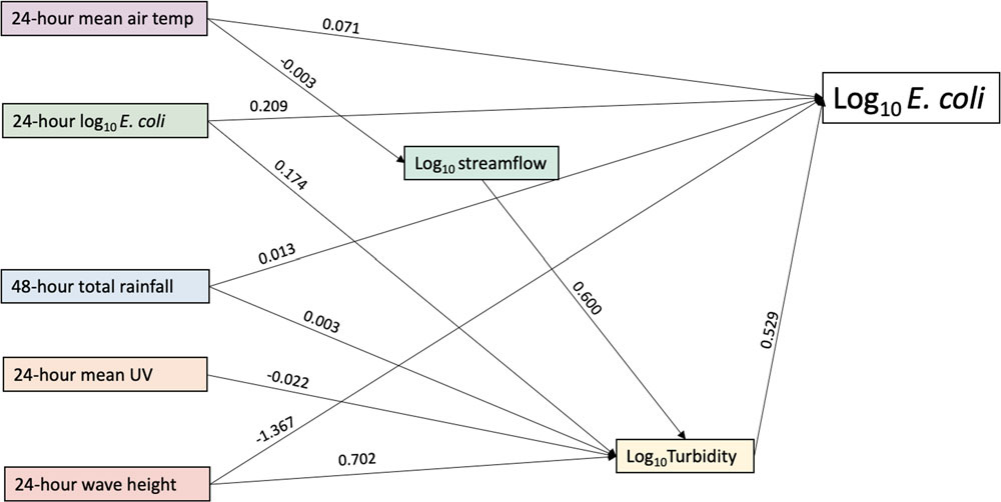
Overall final path model of selected Niagara Region beaches, 2011–2019.

**Fig. 4.**
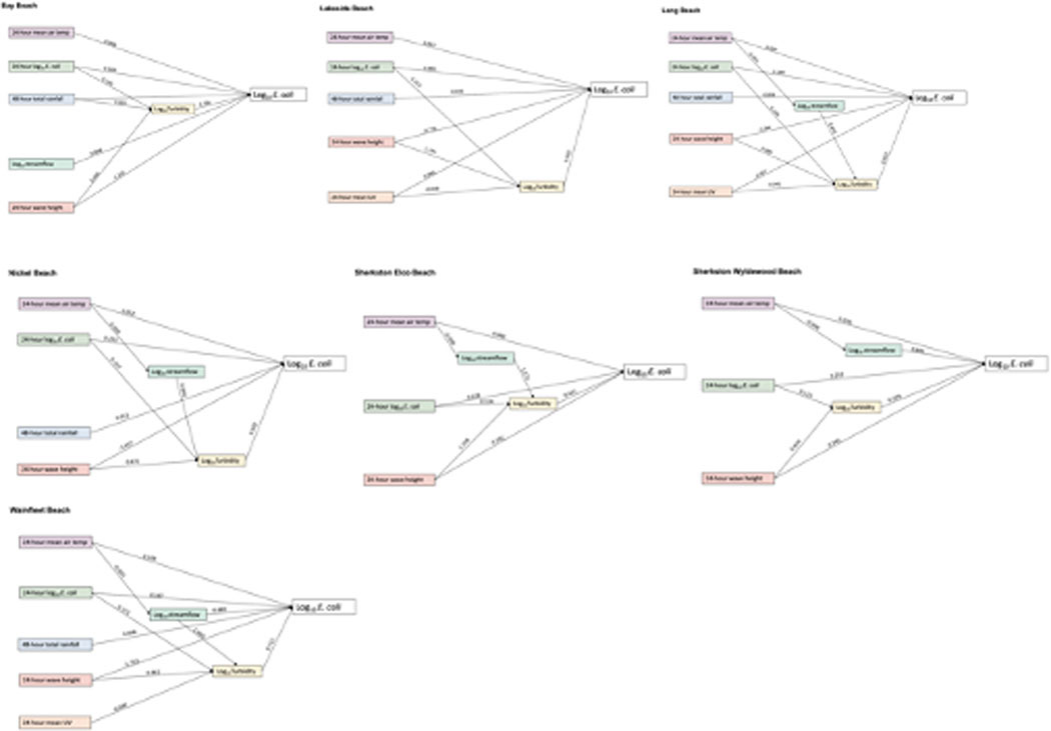
Path diagrams of Niagara Region beaches, 2011–2019.

A total of 3598 observations were linked to previous day *E. coli* concentration and environmental variables, for inclusion in the path analysis (Supplementary Table S2). Final models of the significant overall and beach-specific pathways are presented in path diagrams representing the relationship between the factor or exogenous variable and the mediating variable or with log_10_ *E. coli* directly. In the overall model, model fit indices present a very good model fit with RMSEA = 0.016, chi-square = 0.069, CFI = 0.997 and TLI = 0.992 (Table 1). Model fit indices for the final beach-specific models are presented in Table 2 and suggest very good fit. Estimates of mediation for the indirect effects in the models are presented in Table 2. The overall regional path model presents the results of available observations in all eight participating study beaches, excluding the outfall data (Fig. 3). The data for seven beaches were used to develop beach-specific models and presented in Figure 4. Due to the low sample size at Queen's Royal beach following the linking of observations to antecedent variables, a beach-specific model was not included in the final analysis.

**Table 1.** Model fit of Niagara Region path models, 2011–2019

Model	RMSEA	*χ*^2^	CFI	TLI
Overall	0.016	0.069	0.997	0.992
Bay Beach	0.074	0.096	0.993	0.96
Lakeside Beach	<0.001	0.459	1.00	1.007
Long Beach	<0.001	0.44	1.00	1.001
Nickel Beach	<0.001	0.46	1.00	1.003
Sherkston Elco Beach	<0.001	0.60	1.00	1.028
Sherkston Wyldewood Beach	<0.001	0.83	1.00	1.057
Wainfleet	<0.001	0.91	1.00	1.024

**Table 2.** Estimates of mediation of Niagara Region path models, 2011–2019

	Indirect effects on log_10_ *E. coli* via log_10_ turbidity			Indirect effects on log_10_ turbidity via log_10_ streamflow			Total effects on log_10_ *E. coli*		
Variable	Estimate	s.e.	*P*-value	Estimate	s.e.	*P*-value	Estimate	s.e.	*P*-value
Overall									
24-h mean air temp	−0.001	0.0002	<0.001	−0.002	0.001	<0.001	0.070	0.005	<0.001
48-h total rainfall	0.002	0.0007	0.017				0.015	0.015	<0.001
24-h wave height	0.372	0.036	<0.001				−0.995	−0.996	0.001
24-h log_10_ *E. coli*	0.092	0.008	<0.001				0.301	0.301	<0.001
24-h mean UV	−0.012	0.004	0.010				−0.012	−0.115	<0.001
Log_10_ streamflow	0.318	0.074	<0.001				0.318	0.318	<0.001
Bay Beach									
48-h total rainfall	0.005	0.002	0.040				0.021	0.005	<0.001
24-h wave height	0.264	0.095	0.005				0.264	−0.100	<0.001
24-h log_10_ *E. coli*	0.102	0.022	<0.001				0.102	0.291	<0.001
Lakeside Beach									
24-h log_10_ *E. coli*	0.127	0.021	<0.001				0.212	0.039	<0.001
24-h wave height	0.599	0.149	<0.001				−0.177	0.295	0.549
24-h mean UV	−0.030	0.012	0.017				0.010	0.025	<0.697
Long Beach									
24-h mean air temperature	−0.001	0.001	0.098	−0.002	0.001	0.095	0.096	0.014	<0.001
24-h wave height	0.581	0.094	<0.001				−0.713	0.183	<0.001
24-h log_10_ *E. coli*	0.118	0.019	<0.001				0.306	0.042	<0.001
24-h mean UV	−0.024	0.011	0.035				−0.086	0.026	0.001
Log_10_ streamflow	0.507	0.186	0.006				0.507	0.186	0.006
Nickel Beach									
24-h mean air temperature	−0.002	0.001	0.065	−0.003	0.001	0.062	0.050	0.012	<0.001
24-h wave height	0.516	0.088	<0.001				−1.141	0.180	<0.001
24-h log_10_ *E. coli*	0.063	0.017	<0.001				0.316	0.040	<0.001
Log_10_ streamflow	0.410	0.177	0.020				0.411	0.177	0.020
Sherkston Elco Beach									
24-h mean air temperature	−0.003	0.002	0.057	−0.008	0.004	0.045	0.057	0.020	0.005
24-h wave height	0.534	0.136	<0.001				−1.758	0.303	<0.001
24-h log_10_ *E. coli*	0.048	0.021	0.026				0.277	0.060	<0.001
Log_10_ streamflow	0.536	0.211	0.011				0.536	0.211	0.011
Sherkston Wyldewood									
24-h mean air temperature	−0.006	0.004	0.152				0.064	0.021	0.002
24-h wave height	0.442	0.146	0.002				−1.903	0.324	<0.001
24-h log_10_ *E. coli*	0.059	0.026	0.022				0.312	0.062	<0.001
Wainfleet Beach									
24-h mean air temperature	−0.001	0.001	0.311	−0.006	0.003	0.044	0.108	0.014	<0.001
24-h wave height	0.683	0.109	<0.001				−1.07	0.198	<0.001
24-h log_10_ *E. coli*	0.122	0.021	<0.001				0.288	0.041	<0.001
24-h mean UV	−0.028	0.014	0.040				−0.071	0.029	0.013
Log_10_ streamflow	1.400	0.243	<0.001				0.511	0.443	0.249

Turbidity had a significant and positive direct effect on *E. coli* and was an important mediator, both overall and in all beach-specific models. In the overall model, all environmental factors had a significant indirect effect on *E. coli* via turbidity (Table 2). Increased log_10_ streamflow, which did not have a direct effect on *E. coli,* did have a positive effect on turbidity and therefore a total effect on *E. coli* of 0.318. Turbidity was the mediator for significant indirect effects (Fig. 4) in all beach models. Overall and at Sherkston Elco Beach and Wainfleet Beach, temperature was not directly associated with log_10_ turbidity; however, an increase in previous day mean temperature had a significant negative effect on streamflow and therefore an indirect effect on log_10_ turbidity. A similar pathway was observed at Long Beach and Nickel Beach; however, the indirect effects were not significant (Table 2).

Cumulative rainfall in the preceding 48 h had a positive direct effect on *E. coli,* overall and at all beaches except Sherkston Elco and Sherkston Wyldewood. In the overall model, in addition to its direct effect, rainfall had a statistically significant indirect effect via its positive effect on turbidity (*b* = 0.001, *P* = 0.017), for a total effect of (*b* = 0.002, *P* ≤ 0.001). A similar direct and significant indirect pathway was identified at Bay Beach, while at Lakeside Beach, Long Beach, Nickel Beach and Wainfleet Beach, rainfall had a direct relationship with *E. coli* but no significant indirect pathway via turbidity.

In all models, average wave height in the previous 24 h was positively associated with turbidity and therefore had a positive and statistically significant indirect effect on *E. coli.* Interestingly, however, it had a negative direct effect on *E. coli.* This was consistently reported across beach-specific models as well. Despite the positive indirect effect through turbidity, the higher magnitude negative direct effect resulted in a significant negative total effect of average wave height on *E. coli*.

Overall, previous day air temperature had a direct and positive effect on *E. coli* (*b* = 0.071, *P* ≤ 0.001); however, it had a negative indirect effect via streamflow (*b* = −0.002, *P* ≤ 0.001) and via turbidity (*b* = −0.001, *P* ≤ 0.001). Given the higher magnitude of positive direct effect, total effect of air temperature on *E. coli* was positive. While similar pathways were present at Long Beach, Nickel Beach, Sherkston Elco Beach and Wainfleet Beach, indirect effects via turbidity were not significant. At Sherkston Wyldewood, the indirect path of mean air temperature on *E. coli* via streamflow was not statistically significant. At Bay Beach and Lakeside Beach, the effect of previous day temperature on *E. coli* was direct and positive.

There was consistency in how previous day *E. coli* presented across all models. An increase in value was associated with *E. coli* via a significant positive direct effect and a positive indirect effect via turbidity. Previous day UV had a negative and indirect effect on *E. coli* via turbidity (*b* = −0.012, *P* = 0.010) in the overall model. A similar pathway is observed at Wainfleet Beach, with only negative indirect effects on *E. coli* via log_10_ turbidity. At Lakeside and Long Beach, the effects on *E. coli* were both direct and indirect via turbidity.

As previously described, streamflow was a mediator for previous day air temperature via turbidity, overall and in four out of seven beach models. Direct effects on *E. coli*, in addition to indirect effects, were only identified at Sherkston Wyldewood Beach and Wainfleet Beach. At Wainfleet Beach, the path model describes a significant and positive indirect effect via turbidity (*b* = 1.400, *P* ≤ 0.001); however, the total effect of streamflow was not significant (0.511, *P* = 0.249), suggesting only partial mediation by turbidity.

### Outfall models

**Fig. 5.**
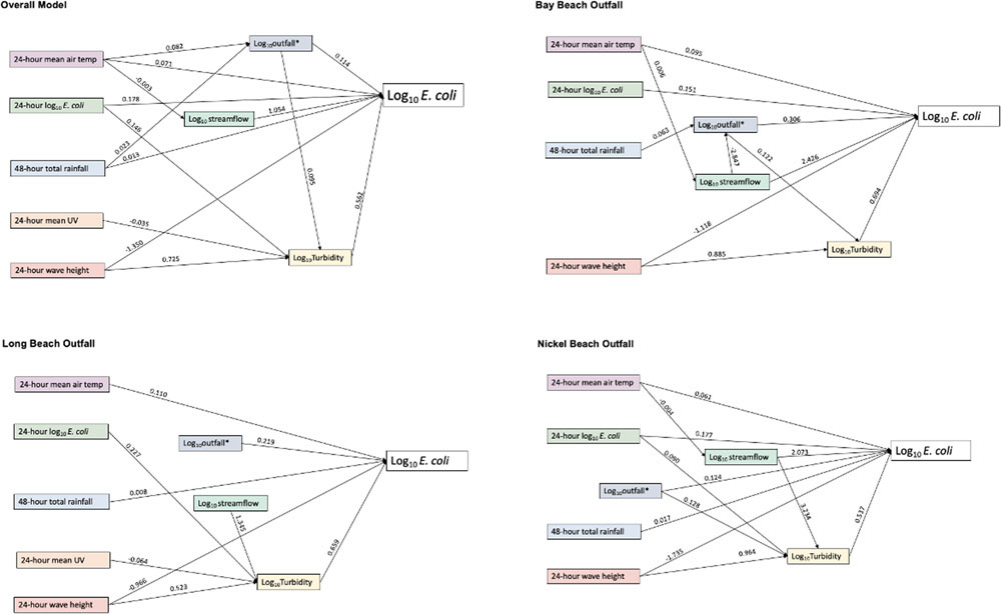
Outfall path diagrams of Niagara Region beaches, 2011–2019.

A total of 738 observations were included in the outfall path analysis (Supplementary Table S2). The overall model is presented in Figure 5 and includes data from all four beaches that collected weekly outfall data. We remind here that due to low sample size, a beach-specific outfall path analysis model was not developed for Queen's Royal Beach. The three beach-specific models are presented in Figure 5. Model fit indices suggest an excellent fit for the overall model and Long Beach model; however, poor fit is observed at Bay Beach and Nickel Beach (Table 3).

**Table 3.** Model fit of Niagara Region outfall path models, 2011–2019

Model	RMSEA	*χ*^2^	CFI	TLI
Overall	0.045	0.045	0.976	0.937
Bay Beach	0.077	0.006	0.951	0.892
Long Beach	0.000	0.542	1.00	1.020
Nickel Beach	0.086	0.008	0.835	0.840

Overall, the addition of the outfall variables resulted in an additional significant mediated pathway for previous day air temperature (*b* = 0.002, *P* = 0.004) and rainfall (*b* = 0.002, *P* ≤ 0.001) (Table 4). In addition to the direct effect of outfall on *E. coli*, there was a positive indirect effect via turbidity (*b* = 0.114, *P* ≤ 0.001) (Table 4). At Long Beach and Nickel Beach, outfall did not mediate the effect of other factors but instead had a positive direct effect, or indirect effect via turbidity (Nickel Beach). At Bay Beach, mean air temperature had a direct effect on *E. coli* and several indirect pathways via streamflow, which then also had pathways through outfall and turbidity. The total effect of mean temperature in the Bay Beach model was 0.103 (*P* ≤ 0.001) (Table 4). Rainfall only had indirect pathways via outfall and then via turbidity for a total positive effect of 0.022 (*P* ≤ 0.001) (Table 4). Similar to Bay Beach, mean temperature at Nickel Beach had a direct effect on *E. coli* and an indirect effect via streamflow, which then had paths to *E. coli* or via turbidity. Total effects were 0.048 (*P* = 0.029).

**Table 4.** Estimates of mediation of Niagara Region outfall path models, 2011–2019

	Indirect effects on log_10_ *E. coli* via log_10_ turbidity			Indirect effects on log_10_ turbidity via log_10_ streamflow			Indirect effects on log_10_ turbidity via log_10_ outfall			Total effects on log_10_ *E. coli*		
Variable	Estimate	s.e.	*P*-value	Estimate	s.e.	*P*-value	Estimate	s.e.	*P*-value	Estimate	s.e.	*P*-value
Overall												
24-h mean air temperature	0.009	0.003	0.002	0.002	0.002	0.004				0.091	0.046	<0.001
48-h total rainfall	0.004	0.0009	<0.001	0.002	0.0005	<0.001				0.013	0.003	<0.001
24-h wave height	0.407	0.086	<0.001							−0.943	0.194	<0.001
24-h log_10_ *E. coli*	0.082	0.016	<0.001							0.260	0.371	<0.001
24-h mean UV	−0.019	0.009	0.033							−0.020	0.009	0.033
Log_10_ outfall	0.114	0.025	<0.001							−0.020	0.009	0.033
Bay Beach												
24-h mean air temp	0.008	0.005	0.103	−0.002	0.001	0.039	−0.017	0.007	0.015	0.103	0.017	<0.001
48-h total rainfall	0.022	0.005	<0.001	−0.008	0.002	<0.001	0.005	0.002	0.021	0.022	0.005	<0.001
24-h wave height	0.614	0.153	<0.001							−0.504	0.306	0.099
Log_10_ streamflow	−1.112	0.377	0.003							1.314	0.727	0.071
Log_10_ outfall	0.085	0.024	<0.001	−0.348	0.142	0.014				0.349	0.046	<0.001
Long Beach												
24-h wave height	0.344	0.186	0.064							−0.621	0.364	0.088
24-h log_10_ *E. coli*	0.150	0.038	<0.001							0.150	0.039	<0.001
24-h mean UV	−0.042	0.022	0.052							−0.119	0.046	0.010
Log_10_ streamflow	0.886	0.417	0.033							0.886	0.417	0.033
Nickel Beach												
24-h mean air temp	−0.014	0.006	0.024	−0.012	0.005	0.016				0.048	0.022	0.029
24-h wave height	0.518	0.142	<0.001							−1.217	0.312	<0.001
24-h log_10_ *E. coli*	0.048	0.025	0.050							0.225	0.065	0.001
Log_10_ streamflow	1.736	0.472	<0.001							3.808	1.029	<0.001
Log_10_ outfall	0.069	0.023	0.003							0.193	0.055	<0.001

## Discussion

While previous studies have examined the factors influencing inland freshwater recreational beach quality, this study used a novel approach in applying path analysis to explore the relationships between environmental factors and their association with *E. coli*. The Niagara Region provides a unique opportunity to explore these dynamics at beaches located along two of the Great Lakes in one of Canada's top tourist destinations, with approximately 14 million tourist visiting the region annually [19]. We used the water quality data, as captured through the measurement of geometric mean of *E. coli*, to explore the overall and beach-specific pathways at seven popular beaches in the region. Exploratory models also aimed to account for a potential annual effect by including ‘year’ as a random effect; however, this did not have a significant impact on the model. The final beach-specific models demonstrate heterogeneity in the significant pathways across the beaches. While some pathways and factors were consistently significant across study sites, there were some notable trends and differences.

An increasing number of studies present a strong relationship between turbidity values and FIB concentration [8, 20, 21]. Suspended particles in the water may shield pathogens from environmental stressors such as UV radiation penetration [3]. In addition, there is increasing evidence that a significant portion of pathogens are associated with particles [22]. A study by Lawrence [8] near Atlanta, Georgia found that that in 34–42% of surface water samples *E. coli* were attached to particles. In our study, turbidity was a consistently important variable in the overall and beach-specific models as having both a direct and positive relationship with *E. coli* concentration and as a mediator for other environmental factors. In the overall model it was a mediator for the effect of all environmental factors. Our results suggest that turbidity is an important factor and mediator in the pathways between environmental factors and *E. coli* concentrations, which needs to be considered and examined further as part of public health strategies. Based on our results, we recommend that public health authorities in Ontario and elsewhere collect turbidity measurements on-site during routine beach water sampling.

Heavy rainfall has been associated with increased microbial concentrations in beach waters [23]. While this analysis did not specifically examine heavy rainfall (e.g. 95th percentile), which has been associated with increased *E. coli* levels [24, 25], it identified increased rainfall in the preceding 48 h had a direct and indirect effect on *E. coli* in 5/7 beaches. Antecedent rainfall was associated with increased *E. coli* concentrations and increased turbidity in the overall and Bay Beach models; however, in the subset outfall data model, rainfall was no longer significant and was instead replaced by outfall *E. coli* concentration, which was associated with both variables. This suggests that rainfall's effect could at least partially be due to outfall levels. Storm water runoff from impervious surfaces has been suggested to be the most important pollution source causing beach closures [25]. Many urban beaches will be automatically posted as unsafe for swimming following rainfall and increased runoff, without water sampling results [25, 26]. Interestingly in 2/7 beaches (Sherkston Elco and Sherkston Wyldewood Beaches), rainfall was not a significant pathway in the models. These findings could suggest that the presence of heavy outfall water flow could be considered to be an indicator of poor water quality conditions.

Overall and at most beaches, stream discharge was indirectly related to *E. coli* concentration via its positive effect on turbidity. This suggests that streamflow may increase turbidity, which was consistently associated with increased *E. coli* concentrations. At Bay Beach, we found there was a positive direct effect on *E. coli* concentration, not mediated by turbidity. Of the beaches examined, this study site was closest in proximity to the start of the Niagara River on the Lake Erie side, which features a strong northward flow through the powerful Niagara Falls. This may be a possible explanation for the important direct effect of streamflow at this location. As presented in the conceptual path diagram (Fig. 3), we expected total rainfall in the previous 48 h to have an effect on streamflow. In an analysis of stormwater samples throughout the duration of storms, it was found that *E. coli* concentration increased during a rain storm were highest in the early stages of a storm [22]. Interestingly, this pathway was consistently not statistically significant across all models; however, as previously described, this study did not examine heavy rainfall specifically, instead only antecedent total rainfall and perhaps this measure does not capture the effect of heavy rain on streamflow specifically.

Research focusing on the effect of UV irradiation on *E. coli* has been mostly limited to the laboratory scale [27]. UV irradiance results in damage to microbial pathogens and is a common method for drinking water treatment [27]. In a laboratory study specifically examining the effect on *E. coli* concentrations, researchers found that exposure to UV light decreased the number of bacterial colonies formed [28]. As previously described, increased turbidity could prevent penetration of UV light and therefore decrease its ability to have an effect on *E. coli* concentrations in water [3]. We used the previous day UV index as water samples are collected early in the morning and have not yet been subjected to extensive UV irradiance on the day of sampling. Research also suggests that increased UV exposure is important for effective treatment [7, 28]. Interestingly, increased UV index had a negative indirect effect on *E. coli* concentration via its negative relationship with turbidity.

Wave activity has been associated with resuspension of bacteria from sediments and therefore elevated *E. coli* concentrations in beach water; however, we did not find this relationship in our models [26]. In contrast with our conceptual diagram, increased wave height was interestingly inversely associated with *E. coli* levels. We did, however, identify that wave height was consistently positively associated with turbidity, which is consistent with the literature, therefore having a positive indirect effect on *E. coli* via that pathway. Due to the greater negative direct effect, the total effect of wave height was negative in most models. Due to collinearity issues, we did not include wind speed as a variable in the models; however, we hypothesised that increased wave height was a proxy for increased wind activity. Important to note is the far offshore location of the buoys used to measure this variable, which may not reflect wave activity at the shoreline. More importantly, strong offshore energy could result in increased water circulation through stronger currents, therefore resulting in increased flushing of shoreline bacteria [29]. In addition, higher energy waters reduce the opportunity for sediment buildup, which are known to provide a habitat for bacteria survival [29].

Temperature is an important factor influencing *E. coli* survival and growth [30]. We examined the relationship of *E. coli* concentrations with air temperature, excluding water temperature due to collinearity issues. Air temperature had a positive direct relationship with *E. coli* concentrations, overall and in all beach models. Several studies have described an inverse relationship between air temperature and streamflow as it can result in marked declines in streamflow [31]. These findings are consistent with the relationships identified in our models. Increased air temperature had a negative effect on streamflow in the overall model as well as five of the beach models.

### Limitations

Given the importance of animal faecal material and microbial loading at beaches, future models could benefit from the inclusion of these data [23]. A microbial source tracking (MST) study conducted in a Toronto beach found that waterfowl was the main source of water contamination and strategies to reduce bird presence were successful in improving water quality [32]. Public health units could consider occasional MST at beaches to improve water quality through targeted approaches [32]. While total rainfall in the previous 48 h was found to have a linear relationship with *E. coli* either directly or via its relationship with turbidity, further exploration by examining rainfall thresholds or heavy precipitation events (e.g. 95th percentiles) could be beneficial to further understand the relationship between rainfall and *E. coli* levels in beach water. Bather load information, which was not available for this analysis, could also be beneficial in informing future models given its suggested association with transference of microorganisms from the sand and sediment to swimming waters and through increased turbidity [3]. Finally, other considerations for future analyses include the addition of information about surrounding cattle and agricultural runoff and proximity to combined sewage systems.

## Conclusion

With almost half a million residents and a large in-flow of tourists during the summer season, the quality of the recreational waters of the Niagara region is of major public health importance. Poor water quality could result in an increased risk of recreational water illness among the thousands of beachgoers that visit the region's many popular beaches. Given the delay in receiving water sampling results due to the culture-based laboratory methods used in the province, a greater understanding of the environmental factors and dynamics for *E. coli* concentration could better guide beach managers in the decision-making and risk communication process. To our knowledge, this is the first application of the path analysis methodology to examine factors of freshwater beach quality. This methodology allowed for the exploration of intervariable relationships via mediation. We identified some clear trends and the importance of some key variables, such as turbidity. We also present the heterogeneity that exists across beaches, despite some clear trends and therefore the need for consideration of site-specific factors when evaluating beach quality. The importance of these results extends beyond the Niagara Region and could be applied to other inland freshwater beaches.

## Data Availability

The environmental data that support the findings of this study are available online from Environment and Climate Change Canada historical data (https://climate.weather.gc.ca/historical_data/search_historic_data_e.html) and the United States NOAA National Weather Service Climate Prediction Centre (https://www.cpc.ncep.noaa.gov/products/stratosphere/uv_index/uv_annual.shtm). *E. coli* data can be publicly accessed online on Niagara Region Open Data (https://niagaraopendata.ca/).

## Supplementary material

For supplementary material accompanying this paper visit http://dx.doi.org/10.1017/S0950268821002120. click here to view supplementary material
