# Supplementary material for: Environmental factors associated with freshwater recreational water quality in Niagara Region, Ontario, Canada: A path analysis
Source: Epidemiol Infect. 2021 Sep 22;149:e217. doi: 10.1017/S0950268821002120 (PMC8527742; doi:10.1017/S0950268821002120)
Supplement: Supplementary file 1 [file S0950268821002120sup001.docx]

**Epidemiology and Infection**

**Environmental factors associated with freshwater recreational water quality in Niagara Region, Ontario, Canada: A Path Analysis**

J. Johanna Sanchez, Ian Young, Cole Heasley, Jeremy Kelly, Anthony Habjan, Ryan Waterhouse, and Jordan Tustin

**Table S1. Summary of linked environmental stations at each beach, Niagara Region, 2011-2019**

| **Beach Name** | **Lake** | **Weather Station** | **Water Level Station** | **Buoy** | **UV** | **Streamflow** |
| --- | --- | --- | --- | --- | --- | --- |
| Bay Beach (Crystal) | Erie | Fort Erie | 02HA017 (Erie) | 45142 | Buffalo | Niagara River |
| Lakeside Beach | Ontario | Grimsby Mountain | 02HA018 (Ontario) | 45159 | Buffalo | Welland canal |
| Long Beach | Erie | Port Colborne | 02HA017 (Erie) | 45142 | Buffalo | Welland canal |
| Nickel Beach | Erie | Port Colborne | 02HA017 (Erie) | 45142 | Buffalo | Welland canal |
| Queen's Royal Beach | Ontario | Grimsby Mountain | 02HA018 (Ontario) | 45159 | Buffalo | Niagara River |
| Sherkston Elco Beach | Erie | Port Colborne | 02HA017 (Erie) | 45142 | Buffalo | Welland canal |
| Sherkston Wyldewood Beach | Erie | Port Colborne | 02HA017 (Erie) | 45142 | Buffalo | Welland canal |
| Wainfleet Lake Erie Public Access Beach | Erie | Port Colborne | 02HA017 (Erie) | 45142 | Buffalo | Welland canal |

**Table S2. Number of observations at each beach, Niagara Region, 2011-2019**

| **Beach Name** | **Total observations** | **No. of linked observations** | |
| --- | --- | --- | --- |
|  |  | **Path analysis** | **Outfall path analysis** |
| Total | 5149 | 3589 | 738 |
| Bay Beach (Crystal) | 843 | 617 | 248 |
| Lakeside Beach | 709 | 576 | N/a |
| Long Beach | 794 | 592 | 185 |
| Nickel Beach | 844 | 647 | 254 |
| Queen's Royal Beach^a^ | 286 | 49 | 49 |
| Sherkston Elco Beach | 444 | 261 | N/a |
| Sherkston Wyldewood Beach | 439 | 258 | N/a |
| Wainfleet Lake Erie Public Access Beach | 790 | 589 | N/a |

**^a^***Beach-specific models were not generated for Queen’s Royal due to low sample size.*

*N/a = outfall data not collected at these beaches.*

**Table S3. Annual geometric means at Niagara Region beaches, 2011-2019**

| **Beach** | **Overall mean** | **2011** | **2012** | **2013** | **2014** | **2015** | **2016** | **2017** | **2018** | **2019** |
| --- | --- | --- | --- | --- | --- | --- | --- | --- | --- | --- |
| Overall | 113 (±184) | 104 (±167) | 88 (±158) | 147 (±221) | 146 (±210) | 91 (±159) | 103 (±167) | 91 (±152) | 101 (±163) | 132 (±213) |
| Bay Beach (Crystal) | 116 (±191) | 88 (±138) | 83 (±139) | 148 (±228) | 178 (±249) | 72(±117) | 81 (±145) | 118 (±192) | 106 (±157) | 184 (±268) |
| Lakeside Beach | 97 (±157) | 116 (±223) | 64 (±101) | 158 (±217) | 132 (±184) | 77 (±125) | 96 (±95) | 44 (±38) | 49 (±77) | 49 (±63) |
| Long Beach | 119 (±181) | 102 (±125) | 92 (±166) | 113 (±157) | 118 (±175) | 102 (±170) | 140 (±208) | 123 (±187) | 166 (±214) | 115 (±193) |
| Nickel Beach | 99 (±172) | 129 (±206) | 106 (±193) | 140 (±225) | 132 (±222) | 78 (±124) | 79 (±131) | 49 (±75) | 60 (±74) | 119 (±186) |
| Queen’s Royal Beach | 156 (±232) | 131 (±180) | 122 (±197) | 274 (±298) | 194 (±268) | 96 (±171) | 127 (±242) | 97 (±138) | 141 (±232) | 134 (±214) |
| Sherkston Elco Beach | 101 (±170) | 99 (±143) | 63 (±130) | 122 (±207) | 153 (±187) | 28 (±30) | 105 (±189) | 31 (±45) | 62 (±110) | 107 (±209) |
| Sherkston Wyldewood Beach | 100 (±165) | 56 (±71) | 72 (±149) | 117 (±215) | 140 (±186) | 50 (±66) | 59 (±102) | 44 (±48) | 69 (±105) | 140 (±211) |
| Wainfleet Lake Erie Public Access Beach | 129 (±205) | 111 (±136) | 107 (±178) | 144 (±224) | 147 (±226) | 141 (±237) | 124 (±211) | 101 (±154) | 150 (±222) | 135 (±210) |

**Table S4. Total (%) annual beach postings (unsafe for swimming) at Niagara Region beaches, 2011-2019**

| **Beach (No. days)** | **No. of days per season (%)** | | | | | | | | |  |
| --- | --- | --- | --- | --- | --- | --- | --- | --- | --- | --- |
|  | **Overall** | **2011 (108)** | **2012 (105)** | **2013 (108)** | **2014 (108)** | **2015 (121)** | **2016 (108)** | **2017 (110)** | **2018 (106)** | **2019 (107)** |
| Bay Beach (Crystal) | 250 (25%) | 35 (32%) | 14 (13%) | 36 (33%) | 38 (35%) | 18 (15%) | 26 (24%) | 33 (30%) | 20 (19%) | 30 (28%) |
| Lakeside Beach* | 394 (40%) | 46 (43%) | 15 (14%) | 45 (42%) | 40 (37%) | 34 (28%) | 37 (34%) | 85 (77%) | 5 (5%) | 87 (81%) |
| Long Beach | 261 (27%) | 36 (33%) | 21 (20%) | 32 (30%) | 34 (31%) | 29 (24%) | 36 (33%) | 36 (33%) | 26 (25%) | 11 (10%) |
| Nickel Beach | 235 (24%) | 44 (41%) | 21 (20%) | 26 (24%) | 36 (33%) | 30 (25%) | 35 (32%) | 14 (13%) | 7 (7%) | 22 (21%) |
| Queen’s Royal Beach | 295 (30%) | 47 (44%) | 33 (31%) | 58 (54%) | 51 (47%) | 25 (21%) | 24 (22%) | 28 (25%) | 14 (13%) | 15 (14%) |
| Sherkston Elco Beach | 182 (19%) | 35 (32%) | 14 (13%) | 31 (29%) | 39 (36%) | 7 (6%) | 28 (26%) | 7 (6%) | 9 (8%) | 12 (11%) |
| Sherkston Wyldewood Beach | 178 (18%) | 20 (19%) | 18 (17%) | 26 (24%) | 36 (33%) | 18 (15%) | 14 (13%) | 14 (13%) | 10 (9%) | 22 (21%) |
| Wainfleet Lake Erie Public Access Beach | 275 (28%) | 37 (34%) | 24 (23%) | 33 (31%) | 31 (29%) | 36 (30%) | 32 (30%) | 35 (32%) | 25 (24%) | 22 (21%) |

******High water levels at Lakeside Beach resulted in extensive beach closure in 2017 and 2019 due to flooding or debris.*

**Table S5. Reasons for posting as unsafe for swimming at Niagara Region beaches, 2011-2019**

| **Reason** | **2011** | **2012** | **2013** | **2014** | **2015** | **2016** | **2017** | **2018** | **2019** |
| --- | --- | --- | --- | --- | --- | --- | --- | --- | --- |
| *E.coli* levels | 283 | 157 | 281 | 301 | 197 | 231 | 174 | 108 | 132 |
| Algae | 0 | 0 | 6 | 4 | 0 | 0 | 0 | 8 | 3 |
| Rainfall | 11 | 2 | 0 | 0 | 0 | 0 | 0 | 0 | 0 |
| Safety* | 0 | 0 | 0 | 0 | 0 | 0 | 0 | 0 | 86 |
| Other* | 6 | 1 | 0 | 0 | 0 | 1 | 74 | 0 | 0 |

******Closures due to high water levels at Lakeside Beach*

**Table S6. Annual environmental predictor values during recreational water sampling season in Niagara Region, 2011-2019**

| **Variable** |  | Total, mean, or median value (±SD) | | | | | | | | |
| --- | --- | --- | --- | --- | --- | --- | --- | --- | --- | --- |
|  | **Overall** | **2011** | **2012** | **2013** | **2014** | **2015** | **2016** | **2017** | **2018** | **2019** |
| Total rainfall (mm) | 2919.3 | 339.7 | 187.0 | 476.5 | 345.8 | 253.3 | 161.2 | 381.2 | 402.4 | 372.3 |
| Mean air temperature (^°^C) | 20.8 (3.7) | 21.3 (3.5) | 22.1 (3.4) | 20.3 (3.9) | 20.0 (3.4) | 19.4 (4.0) | 22.0 (3.7) | 20.3 (2.9) | 22.0 (3.4) | 20.2 (3.6) |
| Mean UV radiation | 7.27 (1.47) | 6.87 (1.91) | 6.98 (1.73) | 6.40 (1.84) | 6.49 (1.53) | 6.62 (1.62) | 6.88 (1.81) | 6.41 (1.79) | 6.66 (1.90) | 6.87 (1.91) |
| Median turbidity (NTU) | 4.6 (9.7) | 5.2 (8.4) | 4.3 (7.4) | 5.0 (6.4) | 5.7 (9.0) | 4.0 (5.2) | 4.4 (6.4) | 4.8 (8.4) | 3.0 (4.2) | 6.5 (12.1) |
| Mean Niagara River discharge (m^3^/s) | 6518 (751) | 6320 (306) | 5547 (276) | 5752 (256) | 6116 (222) | 6551 (415) | 6567 (297) | 7166 (270) | 7168 (285) | 7850 (272) |
| Mean Welland Canal discharge (m^3^/s) | 183 (51.7) | 122 (32.4) | 231 (34.7) | 175 (48.1) | 182 (60.3) | 232 (39.0) | 233 (39.9) | 137 (35.1) | 160 (25.5) | NA |
| Port Colborne Buoy Mean Wave Height (m) | 0.4 (0.28) | 0.4 (0.54) | 0.3 (0.40) | 0.4 (0.54) | 0.3 (0.49) | 0.5 (0.58) | 0.3 (0.45) | 0.2 (0.43) | 0.4 (0.50) | 0.4 (0.48) |
| Mean Grimsby Buoy Wave Height (m) | 0.2 (0.15) | 0.3 (0.36) | 0.2 (0.27) | 0.2 (0.27) | 0.3 (0.36) | 0.2 (0.36) | 0.2 (0.34) | 0.1 (0.25) | 0.3 (0.36) | 0.3 (0.37) |

**Figure S1. Conceptual Path Diagram**

**Outfall was only included in subset models for beaches where it was collected*
